# Association of self-reported sleep duration and quality with BaPWV levels in hypertensive patients

**DOI:** 10.1038/s41440-020-0509-y

**Published:** 2020-07-16

**Authors:** Huan Hu, Huan Li, Xiao Huang, Huihui Bao, Yun Song, Binyan Wang, Chengzhang Liu, Richard Xu, Lishun Liu, Xiaobin Wang, Yong Huo, Xiping Xu, Xiaoshu Cheng, Xianhui Qin, Ping Li

**Affiliations:** 1 Department of Cardiology, the Second Affiliated Hospital of Nanchang University, Nanchang, China; 2 Center for Prevention and Treatment of Cardiovascular Diseases, the Second Affiliated Hospital of Nanchang University, Nanchang, China; 3 National Clinical Research Study Center for Kidney Disease; State Key Laboratory for Organ Failure Research; Renal Division, Nanfang Hospital, Southern Medical University, Guangzhou, China; 4 Beijing Advanced Innovation Center for Food Nutrition and Human Health, College of Food Science and Nutritional Engineering, China Agricultural University, Beijing, China; 5 Institute of Biomedicine, Anhui Medical University, Hefei, China; 6 Shenzhen Evergreen Medical Institute, Shenzhen, China; 7 Krieger School of Arts and Sciences, Johns Hopkins University, Baltimore, USA; 8 Department of Population, Family and Reproductive Health, Johns Hopkins University Bloomberg School of Public Health, Baltimore, USA; 9 Department of Cardiology, Peking University First Hospital, Beijing, China

**Keywords:** Hypertension **·** Sleep duration **·** Sleep quality **·** Arterial stiffness **·** Brachial-ankle pulse-wave velocity

## Abstract

The association between sleep conditions and arterial stiffness remains inconclusive. We aimed to investigate the relationship of sleep duration and quality with brachial-ankle pulse-wave velocity (baPWV) in hypertensive patients. A total of 14,485 hypertensive adults were included in this cross-sectional analysis. Information about sleep duration and quality was obtained via questionnaire. A baPWV level ≥1800 cm/s was defined as indicative of arterial stiffness. Compared with participants with a sleep duration <8 h per day, participants with a sleep duration ≥8 h per day had a significantly higher baPWV level (*β* = 13.7 cm/s; 95% CI: 3.9, 23.5) and a nonsignificantly higher prevalence of arterial stiffness (39.7% vs. 33.0%; OR, 1.08; 95% CI: 0.99–1.19). Similarly, compared with participants with good or medium sleep quality, participants with poor sleep quality had a significantly higher baPWV level (*β* = 16.3 cm/s; 95% CI: 0.1, 32.6) and a nonsignificantly greater prevalence of arterial stiffness (36.6% vs. 35.3%; OR, 1.13; 95% CI: 0.97–1.32). When sleep duration and quality were examined jointly, participants with a sleep duration ≥8 h and/or poor sleep quality had a significantly higher baPWV level (*β* = 14.4 cm/s; 95% CI: 5.3, 23.4) and a greater prevalence of arterial stiffness (38.8% vs. 32.7%; OR, 1.10; 95% CI: 1.01–1.20) than those with a sleep duration <8 h and good/medium sleep quality. In summary, among hypertensive patients, a longer sleep duration (≥8 h per day) and poor sleep quality were associated with higher baPWV levels and a higher prevalence of arterial stiffness.

## Introduction

Arterial stiffness is a subclinical measure of vascular disease, and increased arterial stiffness indicates the deterioration of arterial blood vessel elasticity. To date, arterial stiffness has been reported to be an independent predictor of both atherosclerosis and cardiovascular diseases (CVDs) [1, 2]. Thus, a better understanding of the potential risk factors for increased arterial stiffness may help to prevent the onset of arterial stiffness and related CVDs.

Generally, increased arterial stiffness results from the deterioration of vascular wall structure and function. Previous studies have suggested that chronic sleep fragmentation and sleep restriction may be involved in the progression of endothelial dysfunction and structural vascular changes [3, 4]. Thus, there may be a link between sleep disturbances and arterial stiffness. In fact, several previous studies have investigated the association between abnormal sleep conditions and arterial stiffness, but they have reported inconsistent findings [5–9]. Therefore, the relationship between sleep conditions and arterial stiffness remains inconclusive.

Hypertension is an important risk factor for CVDs and chronic kidney diseases [10, 11]. Hypertension is usually accompanied by the deterioration of sleep quality and increased arterial stiffness [12, 13]. To date, few studies have been conducted to explore the association between sleep disturbances and arterial stiffness among the general hypertensive population. Brachial-ankle pulse-wave velocity (baPWV), a convenient measurement to assess the stiffness of combined large arteries and peripheral arteries, is widely used as a simple noninvasive measure of arterial stiffness to screen subclinical vascular damage in large populations [14, 15]. A higher baPWV value represents a higher level of arterial stiffness, and an increasing number of studies have been performed using the baPWV level to assess arterial stiffness. Therefore, to fill the above knowledge gap, our current study aimed to investigate the relationship between sleep duration and quality with baPWV levels in hypertensive patients using data from the China Stroke Primary Prevention Trial (CSPPT) at site exit visits.

## Methods

### Study participants and design

All participants of this study were from the CSPPT (URL: https://www.clinicaltrials.gov; Unique identifier: NCT00794885). The methods and major results of the CSPPT [16–19] have been reported previously. Briefly, the CSPPT was a multicenter, randomized, double-blind, controlled trial performed from May 2008 to August 2013 in 32 communities in China. The inclusion criteria were participants aged 45 to 75 years old who had hypertension, defined as seated resting systolic blood pressure (SBP) of 140 mmHg or higher or diastolic blood pressure of 90 mmHg or higher at both the screening and recruitment visits or the use of antihypertensive medication. The major exclusion criteria included a history of physician-diagnosed stroke, myocardial infarction, heart failure, coronary revascularization, or congenital heart disease. The CSPPT showed that a combination of enalapril and folic acid is more effective in reducing the risk of first stroke than enalapril alone among a total of 20,702 Chinese hypertensive patients without major CVDs after a median of 4.5 years of follow-up.

Our current study was a cross-sectional analysis using data from the CSPPT at the site exit visit. Overall, of the 20,702 enrolled participants in the CSPPT, 15,197 participants completed the exit site visit and had baPWV measurements. Of those, 14,485 participants with detailed information about sleep duration and quality and with an ABI measurement ≥0.9 were included in the current study (Supplemental fig. 1). This study was approved by the Ethics Committee of the Institute of Biomedicine, Anhui Medical University, Hefei, China. All participants provided written informed consent.

### Measurement of baPWV

The baPWV of each participant was measured using an automatic noninvasive vascular function analyzer (form PWV/ABI, BP-203RPE; Omron-Colin, Japan) [20, 21]. Briefly, after ≥15 min of rest in the supine position, the bilateral brachial arteries and tibial arteries of each subject were wrapped with four oscillometric cuffs connected to a plethysmographic sensor. A phonocardiogram, electrocardiogram, and pulse-wave as well as the blood pressure at both ankles and the brachia can be recorded with the analyzer. Blood pressure, heart rate (HR), and ABI were measured concurrently with PWV. ABI was reported as the ratio of the ankle SBP to the brachial SBP. The value of baPWV was the propagation distance of the pulse wave divided by the pulse-wave propagation time.

According to previous studies [22, 23], a baPWV ≥1800 cm/s was defined as arterial stiffness in this study.

### Data collection

Each participant was interviewed using a standardized questionnaire designed specifically for the present study by trained research staff. The question about sleep duration was phrased as follows: “How long do you usually sleep every day?”, and the following three responses choices were given: <5, 5–8 and ≥8 h. The question about sleep quality was phrased as “How do you describe your sleep quality?”, and 3 response choices (poor, medium, and good) were provided. The question about smoking status was phrased as “How do you describe your smoking?”, and 3 response choices (never, ever, and current) were provided. The question about alcohol consumption status was phrased as “How do you describe your drinking?”, and 3 response choices (never, ever, and current) were provided. The question about physical activity was phrased as “How do you describe your daily physical activity level?”, and 3 response choices (low, moderate, and high) were provided [24–26].

Seated blood pressure was measured using a mercury manometer using the standard method and appropriately sized cuffs by trained research staff after the subjects had been seated for 10 min. Triplicate measurements on the same arm were taken, with at least 2 min between readings. Each patient’s SBP and DBP were calculated as the mean of the three independent measurements.

### Laboratory assays

Serum fasting glucose, lipids, total homocysteine (tHcy), and creatinine levels were measured using automated clinical analyzers (Beckman Coulter, USA) at the core laboratory of the National Clinical Research Center for Kidney Disease, Guangzhou, China.

### Statistical analysis

Baseline characteristics are presented as the means ± standard deviations (SDs) for continuous variables and proportions for categorical variables according to sleep duration or sleep quality. The differences in population characteristics were compared using ANOVA tests, signed rank tests, or chi-square tests.

The relationships of sleep duration and/or sleep quality with baPWV levels and arterial stiffness (baPWV ≥ 1800 cm/s) were evaluated using linear regression models and logistic regression models, respectively, without and with adjustments for age, sex, body mass index (BMI), triglycerides (TGs), total cholesterol (TC), high-density lipoprotein cholesterol (HDL-C), fasting glucose, tHcy, creatinine, SBP, HR, study treatment group, study centers, smoking status, alcohol consumption, and physical activity. Moreover, any potential factors modifying the relationship were explored by subgroup analyses via the assessments of the following variables, including sex, age (<65 vs. ≥65 years), BMI ( < 24 vs. ≥24 kg/m^2^), SBP ( < 140 vs. ≥140 mmHg), current smoking (yes vs. no), current alcohol consumption (yes vs. no), physical activity (low, moderate, and high), TC (<5.2 vs. ≥5.2 mmol/L), fasting glucose (<6.1 vs. ≥6.1 mmol/L or diabetes), and tHcy levels (<12.2 [median] vs. ≥12.2 μmol/L).

A two-tailed *P* < 0.05 was considered to be statistically significant in all analyses. R software (version 3.4.3, http://www.R-project.org) was used for all statistical analyses.

## Results

### Study participants and baseline characteristics

Overall, the mean age of all participants was 64.4 (SD: 7.4) years, 40.2% of them were men, and the mean baPWV was 1727.4 cm/s (SD, 340.3). The general characteristics of the study participants are presented by sleep duration categories (<5, 5–8 and ≥8 h) in Table 1. Participants with a longer sleep duration tended to be older; were more likely to be male, smokers and alcohol consumers; had higher total homocysteine, creatinine, BP levels, and heart rate levels; and had lower sleep quality and HDL-C levels at baseline.

**Table 1:** Characteristics of participants by sleep duration (*n* = 14,485)^a^

Characteristics	Sleep duration per day			*P* value
	<5 h	5–8 h	≥8 h	
Number	621	8667	5197	
Age (years)	64.8 ± 7.4	63.6 ± 7.3	65.6 ± 7.4	<0.001
Male, No. (%)	187 (30.1)	3402 (39.3)	2240 (43.1)	<0.001
Body mass index (kg/m^2^)	24.6 ± 4.0	25.0 ± 3.7	24.9 ± 3.9	0.020
Enalapril–folic acid group, *n* (%)	304 (49.0)	4320 (49.8)	2590 (49.8)	0.910
Current smoker, *n* (%)	95 (15.3)	1838 (21.3)	1116 (21.5)	0.002
Current alcohol drinker, *n* (%)	97 (16.2)	1913 (22.8)	1195 (24.0)	<0.001
Systolic blood pressure (mmHg)	133.9 ± 17.7	135.2 ± 17.2	135.8 ± 17.6	0.022
Diastolic blood pressure (mmHg)	81.3 ± 10.8	82.2 ± 10.9	81.4 ± 10.8	<0.001
Heart rate (bpm)	76.8 ± 11.5	76.6 ± 11.1	77.2 ± 11.7	0.023
baPWV (cm/s)	1733.1 ± 358.1	1704.9 ± 330.4	1764.3 ± 351.1	<0.001
Physical activity, *n* (%)				<0.001
Low	307 (49.4)	3676 (42.4)	2433 (46.9)	
Moderate	210 (33.8)	3707 (42.8)	2130 (41.0)	
High	104 (16.7)	1277 (14.7)	630 (12.1)	
Sleep quality, *n* (%)				<0.001
Good	56 (9.0)	3076 (35.5)	3174 (61.1)	
Medium	130 (20.9)	4747 (54.8)	1806 (34.8)	
Poor	435 (70.0)	844 (9.7)	217 (4.2)	
**Laboratory results**				
Creatinine (μmol/L)	67.9 ± 23.9	67.9 ± 23.7	70.1 ± 31.6	<0.001
Glucose (mmol/L)	6.3 ± 2.3	6.2 ± 1.9	6.3 ± 2.1	0.001
Total cholesterol (mmol/L)	5.4 ± 1.2	5.3 ± 1.1	5.3 ± 1.1	<0.001
Triglyceride (mmol/L)	1.7 ± 1.5	1.8 ± 1.5	1.8 ± 1.4	0.654
HDL-C (mmol/L)	1.33 ± 0.33	1.28 ± 0.31	1.27 ± 0.32	<0.001
Total homocysteine (μmol/L)	13.2 ± 5.0	13.2 ± 6.5	14.0 ± 8.3	<0.001

*baPWV* brachial-ankle pulse-wave velocity, *HDL-C* high-density lipoprotein cholesterol

^a^Variables are presented as mean ± SD or *n* (%)

Moreover, participants with poor sleep quality tended to be younger; were more likely to be females; had a higher heart rate, HDL-C, and TC levels; and had shorter sleep duration, lower BMI, total homocysteine, and creatinine levels at baseline (Table 2).

**Table 2:** Characteristics of participants by sleeping quality (*n* = 14,485)^a^

Characteristics	Sleep quality			*P* value
	Good	Medium	Poor	
Number	6306	6683	1496	
Age (years)	64.6 ± 7.4	64.2 ± 7.3	63.9 ± 7.4	<0.001
Male, No. (%)	2993 (47.5)	2448 (36.6)	388 (25.9)	<0.001
Body mass index (kg/m^2^)	25.0 ± 3.8	24.9 ± 3.8	24.7 ± 3.8	0.014
Enalapril–folic acid group, *n* (%)	3146 (49.9)	3325 (49.8)	743 (49.7)	0.982
Current smoker, *n* (%)	1496 (23.8)	1333 (20.0)	220 (14.7)	<0.001
Current alcohol drinker, *n* (%)	1594 (26.6)	1383 (21.3)	228 (15.6)	<0.001
Systolic blood pressure (mmHg)	135.4 ± 17.5	135.4 ± 17.2	135.0 ± 17.3	0.677
Diastolic blood pressure (mmHg)	81.8 ± 10.9	81.9 ± 10.8	82.1 ± 11.0	0.713
Heart rate (bpm)	76.5 ± 11.4	77.0 ± 11.3	77.6 ± 11.5	<0.001
baPWV (cm/s)	1728.2 ± 342.7	1724.7 ± 337.3	1736.7 ± 343.7	0.453
Physical activity, *n* (%)				<0.001
Low	2961 (47.0)	2749 (41.2)	706 (47.2)	
Moderate	2445 (38.8)	3067 (45.9)	535 (35.8)	
High	895 (14.2)	862 (12.9)	254 (17.0)	
Sleep duration, *n* (%)				<0.001
<5 h	56 (0.9)	130 (1.9)	435 (29.1)	
5–8 h	3076 (48.8)	4747 (71.0)	844 (56.4)	
≥8 h	3174 (50.3)	1806 (27.0)	217 (14.5)	
**Laboratory results**				
Creatinine (μmol/L)	70.5 ± 31.7	67.6 ± 23.1	66.1 ± 18.1	<0.001
Glucose (mmol/L)	6.3 ± 2.0	6.2 ± 2.0	6.3 ± 2.0	0.595
Total cholesterol (mmol/L)	5.3 ± 1.1	5.3 ± 1.1	5.5 ± 1.1	<0.001
Triglyceride (mmol/L)	1.8 ± 1.4	1.8 ± 1.5	1.8 ± 1.3	0.896
HDL-C (mmol/L)	1.27 ± 0.31	1.28 ± 0.31	1.31 ± 0.32	<0.001
Total homocysteine (μmol/L)	13.9 ± 7.9	13.3 ± 6.7	12.9 ± 5.6	<0.001

*baPWV* brachial-ankle pulse-wave velocity, *HDL-C* high-density lipoprotein cholesterol

^a^Variables are presented as mean ± SD or *n* (%)

### Relationship of sleep duration with baPWV and arterial stiffness

In the multivariate models, compared with participants with a sleep duration of 5–8 h, a significantly higher baPWV level (*β*, 14.0 cm/s; 95% CI: 4.1, 23.8) and a nonsignificantly greater prevalence of arterial stiffness (OR, 1.09; 95% CI: 0.99–1.19) were found in those with a sleep duration ≥8 h (Table 3, Supplemental Table 1).

**Table 3:** The relationship of sleep duration and quality with baPWV levels (*n* = 14,485)

	N	baPWV, cm/s Mean (SD)	Crude model		Model I		Model II		Model III	
			*β* (95% CI)	*P* value	*β* (95% CI)	*P* value	*β* (95% CI)	*P* value	*β* (95% CI)	*P* value
**Sleep duration**										
Three categories										
<5 h	621	1733.1 (358.1)	28.2 (0.6, 55.8)	0.046	1.0 (−24.2, 26.2)	0.939	16.2 (−6.2, 38.6)	0.155	6.6 (−17.8, 30.9)	0.597
5–8 h	8667	1704.9 (330.4)	Ref.		Ref.		Ref.		Ref.	
≥8 h	5197	1764.3 (351.1)	59.4 (47.8, 71.1)	<0.001	24.3 (13.6, 35.0)	<0.001	13.5 (3.9, 23.0)	0.006	14.0 (4.1, 23.8)	0.005
Two categories										
<8 h	9288	1706.8 (332.4)	Ref.		Ref.		Ref.		Ref.	
≥8 h	5197	1764.3 (351.1)	57.5 (46.0, 69.0)	<0.001	24.2 (13.6, 34.8)	<0.001	12.4 (2.9, 21.8)	0.010	13.7 (3.9, 23.5)	0.006
**Sleep quality**										
Three categories										
Good	6306	1728.2 (342.7)	Ref.		Ref.		Ref.		Ref.	
Medium	6683	1724.7 (337.3)	−3.5 (−15.2, 8.2)	0.560	−2.1 (−12.8, 8.6)	0.697	−4.6 (−14.2, 4.9)	0.339	−1.4 (−11.3, 8.4)	0.773
Poor	1496	1736.7 (343.7)	8.6 (−10.6, 27.8)	0.381	10.3 (−7.2, 27.9)	0.250	12.4 (−3.2, 28.0)	0.120	15.5 (−1.7, 37.8)	0.077
Two categories										
Good/Medium	12989	1726.4 (339.9)	Ref.				Ref.		Ref.	
Poor	1496	1736.7 (343.7)	10.4 (−7.8, 28.6)	0.264	11.4 (−5.2, 28.1)	0.177	14.8 (0.1, 29.6)	0.048	16.3 (0.1, 32.6)	0.048

Model I: adjusted for age and sex

Model II: adjusted for age, sex, body mass index, triglyceride, total cholesterol (TC), high-density lipoprotein cholesterol, fasting glucose, total homocysteine (tHcy), creatinine, smoking status, alcohol consumption, physical activity, systolic blood pressure (SBP), heart rate, treatment group, and study centers

Model III: all the variables in Model II plus sleep quality or sleep duration

*baPWV* brachial-ankle pulse-wave velocity, *CI* confidence interval

Due to the similar baPWV levels and arterial stiffness prevalence in participants with sleep durations of <5 and 5–8 h (Table 3, Supplemental Table 1), we combined these two groups into one group called sleep duration <8 h. Compared to participants with a sleep duration <8 h, a higher baPWV level (*β*, 13.7 cm/s; 95% CI: 3.9, 23.5) and a greater prevalence of arterial stiffness (OR, 1.08; 95% CI: 0.99–1.19) were found in those with a sleep duration ≥8 h (Table 3, Supplemental Table 1).

### Relationship of sleep quality with baPWV and arterial stiffness

In the multivariate models, compared to participants with good quality, a higher baPWV level (*β*, 15.5 cm/s; 95% CI: −1.7, 37.8) and a greater prevalence of arterial stiffness (OR, 1.13; 95% CI: 0.96–1.33) were found among those with poor sleep quality (Table 3, Supplemental Table 1).

Due to the similar baPWV levels and arterial stiffness prevalence in participants with good and medium sleep quality (Table 3, Supplemental Table 1), we combined these two groups into one group called the good/medium sleep quality group. Compared with participants with good/medium sleep quality, a higher baPWV level (β, 16.3 cm/s; 95% CI: 0.1, 32.6) and a greater prevalence of arterial stiffness (OR, 1.13; 95% CI: 0.97–1.32) were found in those with poor sleep quality (Table 3, Supplemental Table 1).

### Relationship of combined sleep duration and sleep quality with baPWV and arterial stiffness

Compared with participants with a sleep duration of <8 h and good/medium sleep quality, a higher baPWV level was found in those with sleep duration <8 h and poor sleep quality (*β*, 14.5 cm/s; 95% CI: −1.7, 30.8), sleep duration ≥8 h and good/medium sleep quality (*β*, 12.8 cm/s; 95% CI: 3.0, 22.6), or sleep duration ≥8 h and poor sleep quality (β, 49.5 cm/s; 95% CI: 12.7, 86.3) (Table 4). Accordingly, compared with participants with a sleep duration <8 h and good/medium sleep quality, those with a sleep duration ≥8 h and/or poor sleep quality had a significantly higher baPWV level (*β*, 14.4 cm/s; 95% CI: 5.3, 23.4) (Table 4). Similar results were found for arterial stiffness (Supplemental Table 2).

**Table 4:** The relationship of combined sleep duration and sleep quality with baPWV levels (*n* = 14,485)

Combined groups			baPWV, cm/s Mean (SD)	Crude Model		Model I		Model II	
Sleep duration	Sleep quality	*N*		*β* (95% CI)	*P* value	*β* (95% CI)	*P* value	*β* (95% CI)	*P* value
<8 h	Good/Medium	8009	1703.8 (331.4)	Ref.		Ref.		Ref.	
	Poor	1279	1725.7 (337.9)	21.9 (1.8, 41.9)	0.032	14.1 (−4.2, 32.4)	0.130	14.5 (−1.7, 30.8)	0.080
≥8 h	Good/Medium	4980	1762.7 (350.2)	58.9 (46.9, 70.9)	<0.001	24.7 (13.7, 35.6)	<0.001	12.8 (3.0, 22.6)	0.011
	Poor	217	1802.1 (370.3)	98.3 (52.6, 144.0)	<0.001	59.6 (18.0, 101.3)	0.005	49.5 (12.7, 86.3)	0.008
Two categories (Sleep duration and sleep quality)									
<8 h and Good/Medium		8009	1703.8 (331.4)	Ref.		Ref.		Ref.	
≥8 h and/or Poor^a^		6476	1756.7 (348.8)	52.9 (41.8, 64.0)	<0.001	23.7 (13.5, 33.9)	<0.001	14.4 (5.3, 23.4)	0.002

Model I: adjusted for age and sex

Model II: adjusted for age, sex, body mass index, triglyceride, total cholesterol (TC), high-density lipoprotein cholesterol, fasting glucose, total homocysteine (tHcy), creatinine, smoking status, alcohol consumption, physical activity, systolic blood pressure (SBP), diastolic blood pressure (DBP), heart rate, treatment group, and study centers

*baPWV* brachial-ankle pulse-wave velocity, *CI* confidence interval

^a^Sleep duration ≥8 h and/or poor sleep quality includes the three groups of sleep duration (≥8 h) and good/medium sleep quality, sleep duration (≥8 h) and poor sleep quality, and sleep duration (<8 h) and poor sleep quality

### Stratified analyses by potential effect modifiers

Stratified analyses were performed to further assess the association between combined sleep duration and sleep quality (sleep duration ≥8 h and/or poor sleep quality vs. sleep duration <8 h and good/medium sleep quality) with baPWV and arterial stiffness in various subgroups (Table 5 and Supplemental Table 3). No significant interactions were found in the association in any of the subgroups, including sex, age (<65 vs. ≥65 years), BMI (<24 vs. ≥24 kg/m²), SBP (<140 vs. ≥140 mmHg), current alcohol consumption (no vs. yes), intensity of physical activity (low, moderate, and high), TC (<5.2 vs. ≥5.2 mmol/L), fasting glucose (<6.1 vs. ≥6.1 mmol/L or diabetes) and tHcy (<12.2 [median] vs. ≥12.2 μmol/L) subgroups (all *P* for interactions >0.05).

**Table 5:** Stratified analysis of the association between combined sleep duration and quality (sleep duration ≥ 8 h and/or poor sleep quality *vs*. sleep duration <8 h and good/medium sleep quality) with baPWV levels

Subgroups	*N*	<8 h and good/medium	≥8 h and/or poor^a^	Adjusted *β* (95% CI)^b^	*P* for interaction
		baPWV, cm/s Mean (SD)	baPWV, cm/s Mean (SD)		
Sex					0.089
Male	5829	1693.9 (320.6)	1744.9 (348.6)	26.8 (12.0, 41.5)	
Female	8656	1710.6 (338.5)	1764.4 (348.8)	6.8 (−4.6, 18.3)	
Age, years					0.427
<65	7706	1606.4 (282.3)	1633.3 (292.8)	17.4 (6.1, 28.7)	
≥65	6779	1832.8 (347.2)	1872.9 (357.4)	22.1 (7.0, 37.3)	
Body mass index, kg/m^2^					0.448
<24	6079	1740.5 (346.3)	1783.8 (361.9)	17.6 (2.7, 32.4)	
≥24	8366	1678.6 (318.6)	1735.3 (334.6)	13.0 (1.5, 24.4)	
SBP, mmHg					0.955
<140	8944	1615.5 (289.6)	1666.0 (308.6)	17.7 (6.3, 29.0)	
≥140	5421	1852.3 (345.0)	1904.3 (360.0)	14.4 (−2.5, 31.3)	
Current smoking					0.049
No	11402	1707.7 (336.1)	1758.3 (345.7)	9.3 (−0.8, 19.4)	
Yes	3049	1689.8 (314.3)	1751.9 (361.5)	34.2 (13.7, 54.6)	
Current alcohol drinking					0.130
No	10760	1707.4 (333.9)	1760.0 (346.7)	10.4 (0.2, 20.7)	
Yes	3205	1693.9 (325.1)	1751.1 (358.1)	28.7 (9.4, 47.9)	
Physical activity					0.552
Mild	6416	1757.7 (347.3)	1820.2 (367.1)	16.9 (2.3, 31.5)	
Moderate	6047	1675.5 (319.1)	1714.0 (325.6)	14.2 (0.9, 27.6)	
Severe	2011	1631.4 (294.4)	1660.3 (306.2)	2.9 (−18.8, 24.7)	
TC (mmol/L)					0.098
<5.2	7117	1681.4 (321.9)	1739.1 (347.4)	23.0 (10.4, 35.5)	
≥5.2	7112	1727.9 (341.4)	1774.5 (349.1)	6.9 (−6.1, 19.8)	
Glucose (mmol/L)					0.167
<6.1	9174	1678.2 (320.5)	1733.1 (341.8)	18.8 (7.8, 29.9)	
≥6.1 or diabetes^c^	5072	1753.0 (348.2)	1799.2 (357.5)	6.9 (−8.9, 22.6)	
tHcy (μmol/L)					0.400
<12.2	7159	1663.9 (315.2)	1714.9 (330.4)	10.3 (−1.8, 22.4)	
≥12.2	7165	1746.1 (344.0)	1795.2 (360.1)	18.1 (4.6, 31.6)	

*baPWV* brachial-ankle pulse-wave velocity, *CI* confidence interval

^a^Sleep duration ≥8 h and/or poor sleep quality includes the three groups of sleep duration (≥8 h) and good/medium sleep quality, sleep duration (≥8 h) and poor sleep quality, and sleep duration (<8 h) and poor sleep quality

^b^Each subgroup analysis adjusted, if not stratified, for age, sex, body mass index, triglyceride, total cholesterol (TC), high-density lipoprotein cholesterol, fasting glucose, total homocysteine (tHcy), creatinine, smoking status, alcohol consumption, physical activity, systolic blood pressure (SBP), heart rate, treatment group, and study centers

^c^Diabetes was defined as fasting serum glucose ≥7.0 mmol/L or self-reported use of hypoglycemic agents or insulin, or physician diagnosed diabetes

However, a stronger association of combined sleep duration and sleep quality (sleep duration ≥8 h and/or poor sleep quality *vs*. sleep duration <8 h and good/medium sleep quality) with baPWV was found in participants with a current smoking habit (β: 34.2 cm/s; 95% CI: 13.7, 54.6) than in those without a current smoking habit (β: 9.3 cm/s; 95% CI: −0.8, 19.4; *P*-interaction = 0.049).

## Discussion

Our current study revealed that a longer sleep duration (≥8 h) or poor sleep quality was associated with higher baPWV levels and a greater prevalence of arterial stiffness in the general Chinese hypertensive population. Furthermore, current smoking was a modifier of this association.

To date, the relationship between sleep conditions and arterial stiffness remains inconclusive. Kim et al. [9] performed a large cross-sectional study among an apparently healthy population and found that extreme sleep durations (<7 or ≥7 h) and poor subjective sleep quality were associated with an increased prevalence of higher baPWV. Cao et al. [27], using sleep duration ≥8 h as the reference, reported that there was no significant association between a sleep duration of ≤6 or 6–8 h and arterial stiffness. However, participants with poor sleep quality and a sleep duration ≤6 h were more likely to have arterial stiffness than those with good quality sleep who slept for ≥8 h (OR, 2.59; 95% CI: 1.58–4.24). Yoshioka et al. [5] revealed that a daily sleep duration ≥9 h was associated with elevated values of baPWV only among males in a large-scale Japanese study. Another cross-sectional study performed in Taiwan showed that long (>8 h), but not short (<6 h), sleep duration was associated with a higher risk of increased arterial stiffness in males [6]. The discrepancies among these findings may be due to the difference in study populations or the definitions of sleep duration or quality. Our study provided an opportunity to assess the relationship between sleep duration and sleep quality with arterial stiffness in general hypertension-managed patients, with comprehensive adjustments for a series of important confounders.

With a large sample size of 14,485 hypertensive adults, the present study explored the separate effects of sleep duration and sleep quality on baPWV levels with further adjustment for sleep quality or sleep duration, providing several novel insights into the field of the investigation of sleep and arterial stiffness. First, a sleep duration ≥8 h was positively associated with elevated baPWV levels and increased arterial stiffness in hypertensive patients. Consistently, a recent meta-analysis by Jike et al. [28] including 5,134,036 subjects from 137 prospective cohort studies concluded that a self-reported longer sleep duration was significantly associated with mortality (RR, 1.39; 95% CI, 1.31, 1.47), CVDs (1.25; 1.14, 1.37), stroke (1.46; 1.26, 1.69) and coronary heart disease (1.24; 1.13, 1.37). However, the exact mechanisms behind the relationship of long sleep duration and these health outcomes have not yet been elucidated. Grandner et al. [29] indicated that sleep fragmentation, fatigue, immune functionality, lack of challenges, depression, or underlying disease processes, such as sleep apnea, may be potential mechanisms of the association between long sleep and mortality. The present study found a positive relationship between self-reported longer sleep duration (≥8 h) and higher baPWV levels, providing a potential mechanism linking long sleep duration and poor health outcomes via mediation of arterial stiffness. Irwin et al. [30] indicated that longer sleep durations (>8 h per night), but not shorter sleep durations (<7 h per night), were associated with elevated levels of systemic inflammation markers, which could increase arterial stiffness by enabling vascular fibrosis and smooth muscle cell proliferation [31]. Therefore, the inflammatory pathway may be one of the mechanisms involved in the association between longer sleep duration and arterial stiffness. Our current study failed to find a similar association between short sleep duration and the risk of arterial stiffness, which might be partly due to the difference in study populations and the definition of sleep duration. More studies concerning the association between objectively measured sleep duration and baPWV levels are needed to confirm our findings.

Second, the present study also found a positive association between poor sleep quality and increased baPWV levels in hypertensive patients. Consistently, the study by Osonoi et al. [7], including 724 patients with type 2 diabetes mellitus, revealed that poor sleep quality, assessed by the Pittsburg Sleep Quality Index (PSQI), was associated with increased arterial stiffness. Moreover, the study by Guney et al. [32], including 199 patients with chronic kidney disease, found that participants with poor sleep quality, assessed by the PSQI, had higher PWV levels than those with good sleep quality. However, only 73 subjects with poor sleep quality were included in the study; therefore, the power to identify the association was poor. Recently, the HSCAA cohort study [8], including 306 patients with cardiovascular risks, found that low sleep quality was associated with the progression of arterial stiffness over a 3-year follow-up period, the OR of which was (3.62; 95% CI: 1.04–12.55). However, no specific scientific discussions were available regarding this association among hypertensive patients in these findings. Our results filled the knowledge gap in this field. The exact mechanisms behind the association between sleep quality and arterial stiffness remain inconclusive. Previous studies have suggested that insulin resistance [33], increased sympathetic activity [34] and endothelial dysfunction [35] may be the potential mechanisms underlying this association. Further studies are warranted to identify more potential mechanisms.

Third, we investigated the combined effect of sleep duration and sleep quality on baPWV levels and indicated that longer sleep duration and/or poor sleep quality was associated with increased baPWV levels and arterial stiffness. Moreover, participants with longer sleep durations and poor sleep quality had the highest baPWV levels and prevalence risk of arterial stiffness compared with the other groups. The findings suggest that poor sleep quality may increase the risk of arterial stiffness resulting from longer sleep duration. Regarding the combined effect of sleep duration and sleep quality on the risk of arterial stiffness, the combined effect was not statistically significant in the group with longer sleep duration and poor sleep quality, although the combined risk was the highest (OR, 1.33; 95% CI, 0.94, 1.89) (Supplemental Table 2). This might be due to the small number of participants in the group (*n* = 217). Stratified analysis revealed that the association between combined sleep duration and sleep quality (sleep duration ≥8 h and/or poor sleep quality vs. sleep duration <8 h and good/medium sleep quality) with baPWV was stronger among current smokers. Previous studies have reported that both acute and chronic smoking were positively associated with increased arterial stiffness [36], whereas there was a reversible effect of smoking cessation on arterial stiffness [37]. The exact mechanisms underlying the effect of current smoking on this association remain unclear. A plausible biological explanation may be that abnormal sleep conditions and smoking might share several of the same cellular and molecular mechanisms, including insulin resistance and inflammation, for the pathogenesis of arterial stiffness. Further studies are needed to verify this hypothesis.

Several limitations should not be ignored. First, this was a cross-sectional analysis of the CSPPT and could not clarify the causal relationship between sleep conditions and higher baPWV levels. Second, the present study only explored the association of self-reported sleep conditions, but not objective measures of sleep conditions, with baPWV levels. As several previous studies have reported that there were discrepancies between self-reported and objective measures of sleep conditions when assessing the associations between sleep conditions and hypertension and metabolic syndrome [38, 39], further studies concerning the relationship of objective measures of sleep conditions and baPWV levels are needed to confirm our findings. Finally, the association between sleep conditions and baPWV levels was observed only among treated hypertensive patients in our study, so the findings might not be applicable to other populations.

## Conclusions

In summary, a longer sleep duration (≥8 h) or poor sleep quality was associated with increased baPWV levels and arterial stiffness in hypertensive patients. Maintaining proper sleep duration and good sleep quality may play a positive role in maintaining a relatively low level of arterial stiffness among hypertensive patients. If further confirmed, evaluations of sleep duration and quality along with other known risk factors could help to further identify hypertensive patients at high risk of arterial stiffness.

## Supplementary information

Supplementary Figure 1

Supplemental Table 1

Supplemental Table 2

Supplemental Table 3
