# Supplementary material for: Association of self-reported sleep duration and quality with BaPWV levels in hypertensive patients
Source: Hypertens Res. 2020 Jul 16;43(12):1392–402. doi: 10.1038/s41440-020-0509-y (PMC7671938; doi:10.1038/s41440-020-0509-y)
Supplement: Supplementary file 1 — Supplementary Figure 1 [file 41440_2020_509_MOESM1_ESM.doc]

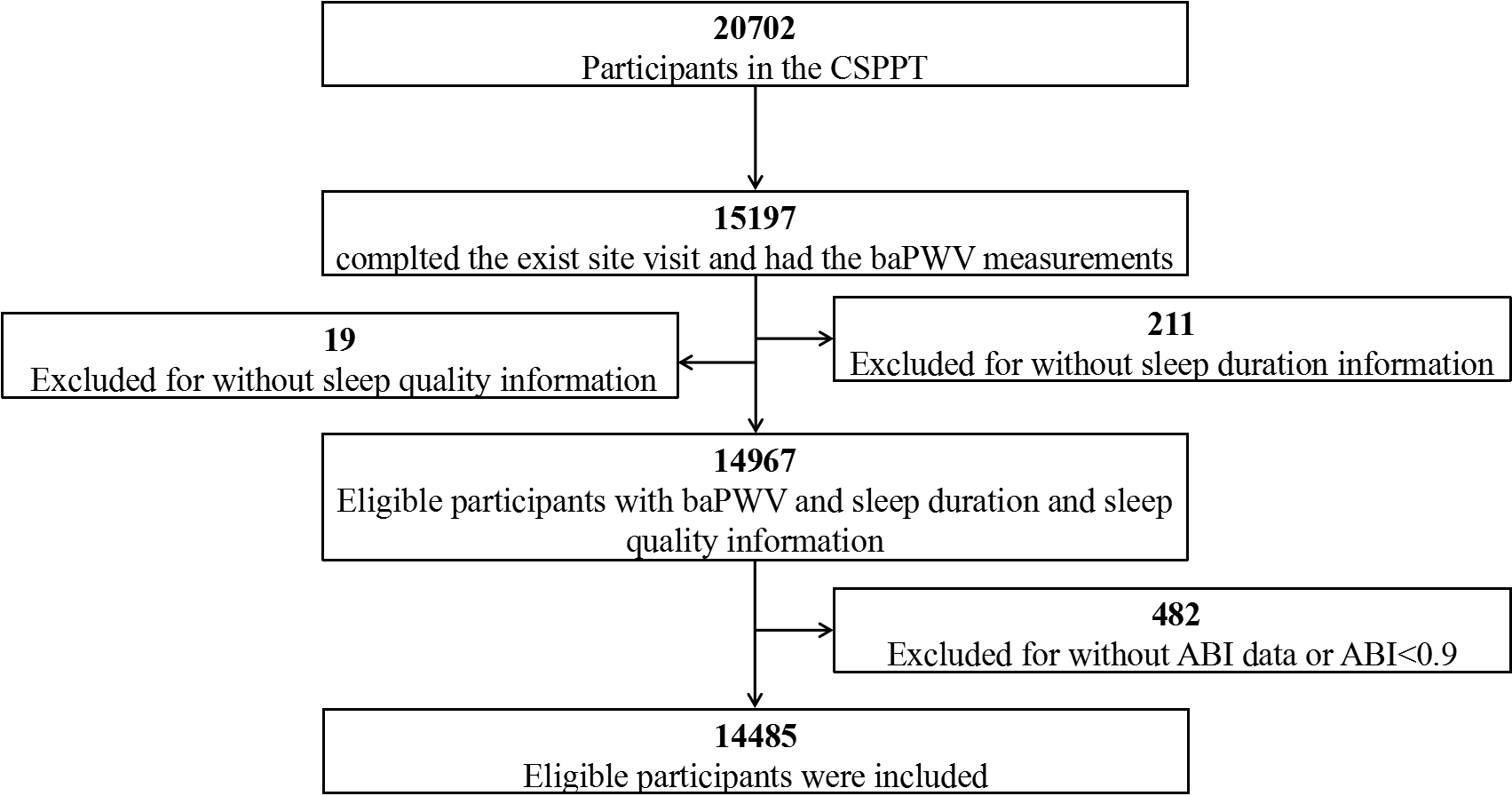


**Supplemental figure 1. Flow chart of the present study population. Abbreviations:** CSPPT, China Stroke Primary Prevention Trial; baPWV, brachial-ankle pulse wave velocity; ABI, ankle brachial index.
