# Supplementary material for: Association of self-reported sleep duration and quality with BaPWV levels in hypertensive patients
Source: Hypertens Res. 2020 Jul 16;43(12):1392–402. doi: 10.1038/s41440-020-0509-y (PMC7671938; doi:10.1038/s41440-020-0509-y)
Supplement: Supplementary file 2 — Supplemental Table 1 [file 41440_2020_509_MOESM2_ESM.doc]

**Supplemental table 1. The relationship of sleep duration and quality with increased arterial stiffness (measured as baPWV ≥1800 cm/s)**

|  | N | Events (%) | Crude model | | Model I | | Model II | | Model III | |
| --- | --- | --- | --- | --- | --- | --- | --- | --- | --- | --- |
|  |  |  | OR (95%CI) | *P* Value | OR (95%CI) | *P* Value | OR (95%CI) | *P* Value | OR (95%CI) | *P* Value |
| **Sleep duration** | | | | | | | | | | |
| *Three categories* | | | | | | | | | | |
| <5 h | 621 | 228 (36.7) | 1.19 (1.01, 1.41) | 0.044 | 1.04 (0.86, 1.24) | 0.697 | 1.20 (0.97, 1.48) | 0.096 | 1.11 (0.88, 1.40) | 0.371 |
| 5-8 h | 8667 | 2840 (32.8) | Ref. |  | Ref. |  | Ref. |  | Ref. |  |
| ≥8 h | 5197 | 2062 (39.7) | 1.35 (1.26, 1.45) | <0.001 | 1.13 (1.05, 1.22) | 0.001 | 1.08 (0.99, 1.18) | 0.084 | 1.09 (0.99, 1.19) | 0.077 |
| *Two categories* | | | | | | | | | | |
| <8 h | 9288 | 3068 (33.0) | Ref. |  | Ref. |  | Ref. |  | Ref. |  |
| ≥8 h | 5197 | 2062 (39.7) | 1.33 (1.24, 1.43) | <0.001 | 1.13 (1.05, 1.22) | 0.002 | 1.07 (0.98, 1.17) | 0.138 | 1.08 (0.99, 1.19) | 0.093 |
| **Sleep quality** | | | | | | | | | | |
| *Three categories* | | | | | | | | | | |
| Good | 6306 | 2234 (35.4) | Ref. |  | Ref. |  | Ref. |  | Ref. |  |
| Medium | 6683 | 2349 (35.1) | 0.99 (0.92, 1.06) | 0.741 | 1.00 (0.93, 1.08) | 0.943 | 0.97 (0.89, 1.06) | 0.507 | 0.99 (0.90, 1.08) | 0.808 |
| Poor | 1496 | 547 (36.6) | 1.05 (0.93, 1.18) | 0.409 | 1.08 (0.95, 1.23) | 0.225 | 1.12 (0.97, 1.30) | 0.119 | 1.13 (0.96, 1.33) | 0.154 |
| *Two categories* | | | | | | | | | | |
| Good/Medium | 12989 | 4583 (35.3) | Ref. |  | Ref. |  | Ref. |  | Ref. |  |
| Poor | 1496 | 547 (36.6) | 1.06 (0.95, 1.18) | 0.327 | 1.08 (0.96, 1.22) | 0.209 | 1.14 (0.99, 1.32) | 0.061 | 1.13 (0.97, 1.32) | 0.110 |

Model I: adjusted for age and sex. Model II: adjusted for age, sex, body mass index, triglyceride, total cholesterol (TC), high-density lipoprotein cholesterol, fasting glucose, total homocysteine (tHcy), creatinine, smoking status, alcohol consumption, physical activity, systolic blood pressure (SBP), ~~diastolic blood pressure (DBP)~~, heart rate, treatment group, and study centers. Model III: all the variables in Model II plus sleep quality or sleep duration.

**Abbreviations:** OR indicates odds ratio; and CI, confidence interval.
