# Supplementary material for: Association of self-reported sleep duration and quality with BaPWV levels in hypertensive patients
Source: Hypertens Res. 2020 Jul 16;43(12):1392–402. doi: 10.1038/s41440-020-0509-y (PMC7671938; doi:10.1038/s41440-020-0509-y)
Supplement: Supplementary file 3 — Supplemental Table 2 [file 41440_2020_509_MOESM3_ESM.doc]

**Supplemental table 2. The relationship of combined sleep duration and sleep quality with arterial stiffness (measured as baPWV ≥1800 cm/s)**

| Combined Groups | | N | Events (%) | Crude Model | | Model I | | Model II | |
| --- | --- | --- | --- | --- | --- | --- | --- | --- | --- |
| Sleep duration | Sleep quality |  |  | OR (95% CI) | *P* Value | OR (95% CI) | *P* Value | OR (95% CI) | *P* Value |
| <8 h | Good/Medium | 8009 | 2617 (32.7) | Ref. |  | Ref. |  | Ref. |  |
|  | Poor | 1279 | 451 (35.3) | 1.12 (0.99, 1.27) | 0.068 | 1.10 (0.96, 1.25) | 0.178 | 1.15 (0.99, 1.35) | 0.069 |
| ≥8 h | Good/Medium | 4980 | 1966 (39.5) | 1.34 (1.25, 1.45) | <0.001 | 1.14 (1.05, 1.23) | 0.002 | 1.08 (0.99, 1.18) | 0.096 |
|  | Poor | 217 | 96 (44.2) | 1.63 (1.25, 2.15) | <0.001 | 1.38 (1.03, 1.86) | 0.031 | 1.33 (0.94, 1.89) | 0.110 |
| *Two categories* (Sleep duration and sleep quality) | | | | | | | | | |
| <8 h and Good/Medium | | 8009 | 2617 (32.7) | Ref. |  | Ref. |  | Ref. |  |
| ≥8 h and/or Poor^*^ | | 6476 | 2513 (38.8) | 1.31 (1.22, 1.40) | <0.001 | 1.14 (1.05, 1.22) | <0.001 | 1.10 (1.01, 1.20) | 0.026 |

Model I: adjusted for age and sex. Model II: adjusted for age, sex, body mass index, triglyceride, total cholesterol (TC), high-density lipoprotein cholesterol, fasting glucose, total homocysteine (tHcy), creatinine, smoking status, alcohol consumption, physical activity, systolic blood pressure (SBP), ~~diastolic blood pressure (DBP)~~, heart rate, treatment group, and study centers. ^*^Sleep duration ≥8 h and/or poor sleep quality includes the three groups of sleep duration (≥8 h) and good/medium sleep quality, sleep duration (≥8 h) and poor sleep quality, and sleep duration (<8 h) and poor sleep quality.

**Abbreviations:** OR indicates odds ratio; and CI, confidence interval.
