# Supplementary material for: Association of self-reported sleep duration and quality with BaPWV levels in hypertensive patients
Source: Hypertens Res. 2020 Jul 16;43(12):1392–402. doi: 10.1038/s41440-020-0509-y (PMC7671938; doi:10.1038/s41440-020-0509-y)
Supplement: Supplementary file 4 — Supplemental Table 3 [file 41440_2020_509_MOESM4_ESM.doc]

**Supplemental table 3. Stratified analysis of the association between combined sleep duration and quality (sleep duration ≥8 hours and/or poor sleep quality *vs.* sleep duration <8 hours and good/medium sleep quality) with arterial stiffness (measured as baPWV ≥1800 cm/s)**

| Subgroups | N | <8 hours and good/medium | | ≥8 hours and/or poor^†^ | Adjusted OR (95%CI)^*^ | *P* for interaction |
| --- | --- | --- | --- | --- | --- | --- |
|  |  | Events (%) | | Events (%) |  |  |
| Sex | | | | | | 0.332 |
| Male | 5829 | 1042 (31.9) | | 964 (37.6) | 1.17 (1.02, 1.34) |  |
| Female | 8656 | 1575 (33.2) | | 1549 (39.6) | 1.06 (0.95, 1.18) |  |
| Age, y | | | | | | 0.456 |
| <65 | 7706 | 937 (20.5) | 749 (23.8) | | 1.19 (1.05, 1.35) |  |
| ≥65 | 6779 | 1680 (48.8) | 1764 (52.9) | | 1.10 (0.99, 1.23) |  |
| Body mass index, kg/m² | | | | | | 0.723 |
| <24 | 6079 | 1201 (36.7) | 1174 (41.9) | | 1.13 (0.99, 1.28) |  |
| ≥24 | 8366 | 1410 (29.9) | 1333 (36.4) | | 1.09 (0.97, 1.22) |  |
| SBP, mmHg | | | | | | 0.648 |
| <140 | 8944 | 1101 (22.1) | | 1102 (27.8) | 1.12 (1.00, 1.25) |  |
| ≥140 | 5421 | 1496 (50.4) | | 1390 (56.6) | 1.09 (0.97, 1.24) |  |
| Current smoking | | | | | | 0.377 |
| No | 11402 | 2063 (33.0) | | 2021 (39.2) | 1.08 (0.98, 1.19) |  |
| Yes | 3049 | 548 (31.3) | | 487 (37.5) | 1.21 (1.00, 1.47) |  |
| Current alcohol drinking | | | | | | 0.078 |
| No | 10760 | 1957 (33.0) | | 1886 (39.0) | 1.05 (0.96, 1.16) |  |
| Yes | 3205 | 570 (31.5) | | 541 (38.7) | 1.29 (1.08, 1.54) |  |
| Physical activity | | | | | | 0.181 |
| Mild | 6416 | 1340 (39.6) | | 1411 (46.5) | 1.10 (0.97, 1.24) |  |
| Moderate | 6047 | 990 (28.6) | | 873 (33.7) | 1.17 (1.03, 1.34) |  |
| Severe | 2011 | 285 (24.5) | | 227 (26.7) | 0.88 (0.69, 1.14) |  |
| TC (mmol/L) | | | | | | 0.328 |
| <5.2 | 7117 | 1216 (30.1) | | 1132 (36.8) | 1.16 (1.02, 1.31) |  |
| ≥5.2 | 7112 | 1358 (35.4) | | 1340 (40.9) | 1.06 (0.94, 1.19) |  |
| Glucose (mmol/L) | | | | | | 0.446 |
| <6.1 | 9174 | 1543 (29.9) | | 1446 (36.0) | 1.13 (1.01, 1.26) |  |
| ≥6.1 or diabetes^#^ | 5072 | 1032 (38.0) | | 1030 (43.7) | 1.06 (0.93, 1.23) |  |
| tHcy (μmol/L) | | | | | | 0.727 |
| <12.2 | 7159 | 1147 (28.0) | | 1036 (33.8) | 1.07 (0.95, 1.22) |  |
| ≥12.2 | 7165 | 1438 (37.7) | | 1453 (43.4) | 1.12 (1.00, 1.26) |  |

^*^Each subgroup analysis adjusted, if not stratified, for age, sex, body mass index, triglyceride, total cholesterol (TC), high-density lipoprotein cholesterol, fasting glucose, total homocysteine (tHcy), creatinine, smoking status, alcohol consumption, physical activity, systolic blood pressure (SBP), ~~diastolic blood pressure (DBP)~~, heart rate, treatment group, and study centers. ^#^Diabetes was defined as fasting serum glucose ≥7.0 mmol/L or self-reported use of hypoglycemic agents or insulin, or physician diagnosed diabetes. ^†^Sleep duration ≥8 h and/or poor sleep quality includes the three groups of sleep duration (≥8 h) and good/medium sleep quality, sleep duration (≥8 h) and poor sleep quality, and sleep duration (<8 h) and poor sleep quality.

**Abbreviations:** OR indicates odds ratio; and CI, confidence interval.
